# Direct evidence of microstructure dependence of magnetic flux trapping in niobium

**DOI:** 10.1038/s41598-021-84498-x

**Published:** 2021-03-08

**Authors:** Shreyas Balachandran, Anatolii Polyanskii, Santosh Chetri, Pashupati Dhakal, Yi-Feng Su, Zu-Hawn Sung, Peter J. Lee

**Affiliations:** 1 Applied Superconductivity Center, NHMFL-FSU, Tallahassee, FL 32309 USA; 2 Thomas Jefferson National Accelerator Facility, Newport News, VA 23606 USA; 3 Oak Ridge National Laboratory, Oak Ridge, TN 37830 USA; 4 Fermi Lab, Batavia, IL 60510 USA

**Keywords:** Superconducting properties and materials, Applied physics, Materials science

## Abstract

Elemental type-II superconducting niobium is the material of choice for superconducting radiofrequency cavities used in modern particle accelerators, light sources, detectors, sensors, and quantum computing architecture. An essential challenge to increasing energy efficiency in rf applications is the power dissipation due to residual magnetic field that is trapped during the cool down process due to incomplete magnetic field expulsion. New SRF cavity processing recipes that use surface doping techniques have significantly increased their cryogenic efficiency. However, the performance of SRF Nb accelerators still shows vulnerability to a trapped magnetic field. In this manuscript, we report the observation of a direct link between flux trapping and incomplete flux expulsion with spatial variations in microstructure within the niobium. Fine-grain recrystallized microstructure with an average grain size of 10–50 µm leads to flux trapping even with a lack of dislocation structures in grain interiors. Larger grain sizes beyond 100–400 µm do not lead to preferential flux trapping, as observed directly by magneto-optical imaging. While local magnetic flux variations imaged by magneto-optics provide clarity on a microstructure level, bulk variations are also indicated by variations in pinning force curves with sequential heat treatment studies. The key results indicate that complete control of the niobium microstructure will help produce higher performance superconducting resonators with reduced rf losses^1^ related to the magnetic flux trapping.

## Introduction

Superconducting radio frequency (SRF) cavities made from high purity niobium (Nb) are now used extensively to accelerate charged particles in nuclear physics research and in the production of intensely high-quality beams for light sources^1^. One of the most important parameters for assessing cavity performance is the quality factor, *Q*_0_, which is defined as *Q*_0_ = *ωU/P*_d_, where omega is the resonant frequency (rad/s), *U* is the stored energy (J) and *P*_d_ is the power dissipated (W) in the cavity to maintaining energy U; current state-of-the-art industrially produced Nb cavities reach remarkably high*Q*_*0*_ values, well above 10^10^ at accelerating gradient *E*_*acc*_ > 20 MV/m^2^ at an operating temperature of ~ 2 K^1^. High *Q*_*0*_ with high accelerating gradients enable more cryogenically efficient and high energy accelerators. However, the achievement of both high *Q*_*0*_ and high *E*_*acc*_ in the same cavity has remained elusive. Beyond SRF accelerator technology, high *Q*_*0*_ SRF quality Nb is beginning to find application in quantum computing devices^3,4^, with the possibility that high Q at very low microwave power at a single photon level may provide a viable approach to increase qubit lifetime^5,6^. Hence, understanding the factors that determine *Q*_*0*_ in SRF Nb is of great interest.

Recent increases in *Q*_*0*_ values obtained with 800 °C heat treatments have been mainly achieved through the development of final processing recipes that modify the Nb surface layer^3^ within the radio frequency (rf) penetration depth. Techniques that have been shown to beneficially alter the Nb surface within the first few nanometers to microns include low temperature baking^7^, high-temperature heat treatments^8^, and diffusion of solutes titanium (Ti)^8^, nitrogen (N)^9^ at high temperatures of 800 –1400 °C. Surface modifications change the nature of surface oxide^10^, hydrogen concentration^11,12^, and create a dirty superconducting layer^13–15^. Promising new results indicate high *Q*_*0*_ at high *E*_*acc*_ up to 40 MV/m is possible by low-temperature N treatments in the range of 120–200 °C^13,16,17^. An increase in *Q*_*0*_ with an initial accelerating gradient (also known as the *Q-*rise behavior) can be produced by these treatments and has been attributed to variations in the nonlinear surface resistance on rf field^18^. The nonlinear dependence has been theoretically explained by changes in the density of states due to a dirty superconducting layer or nanometric surface features of Nb^19–21^. Correlations also exist between the variation in electronic mean free path due to interstitial contamination and the occurrence of a minimum in the Bardeen-Cooper-Schrieffer (BCS)resistance component of surface resistance for an optimal mean free path^22^.

The pursuit of high *Q*_*0*_ values at high *E*_*acc*_ has led to the exploration of issues related to increases in the temperature-independent component of surface resistance, commonly known as residual resistance (*R*_*res*_), due to trapped residual magnetic flux in Nb. The trapping of flux is the result of the inability of Nb to completely expel magnetic flux during cooling of the cavity to below the superconducting state (*T*_*c*_ = 9.2 K). It has been observed that magnetic flux can get trapped in Nb during cavity cool down even in ambient magnetic fields, leading to an increase in overall cavity resistance by a few nΩ/mG of trapped flux. Efficient flux expulsion has been shown to be possible by controllably removing the surface damage^23^ and by enhancing bulk structures with heat treatments^18^. However, there are still significant variations in flux trapping and flux expulsion from Nb cavities that are not well understood, for instance, cavities manufactured from Nb sheet with same material specifications produced by the same vendor, and treated by the same recipe produces cavities that expel flux differently^2^. The increase in residual resistance due to the trapped magnetic field has been studied in several SRF cavities with respect to the starting Nb material^24^, surface preparation^18^, and nitrogen diffusion conditions^25^. Theoretically, a multi-scale collective pinning mechanism is suggested for rf dissipation in SRF cavities^26^, and a recent review indicates pinning possibilities could be related to dislocation structures^27^.

In this manuscript, we address the issue of microstructure influenced flux trapping based on a systematic study of a deformed bi-crystal microstructure undergoing successive heat treatments. We provide direct evidence of grain size variations regarding flux trapping in recrystallized Nb as observed by combining magneto optical imaging (MOI) with electron backscattered diffraction–orientation imaging microscopy (EBSD-OIM). DC magnetization measurements are also used to quantify the flux pinning force in the samples. These results demonstrate that better engineering of the Nb microstructures could lead to reproducibly low flux trapping and consequently provide improved routes for the production of high *Q*_*0*_ for SRF cavities for accelerator and other rf applications.

## Results

### Microstructure of Nb bi-crystal after deformation and subsequent heat treatments

**Figure 1:**
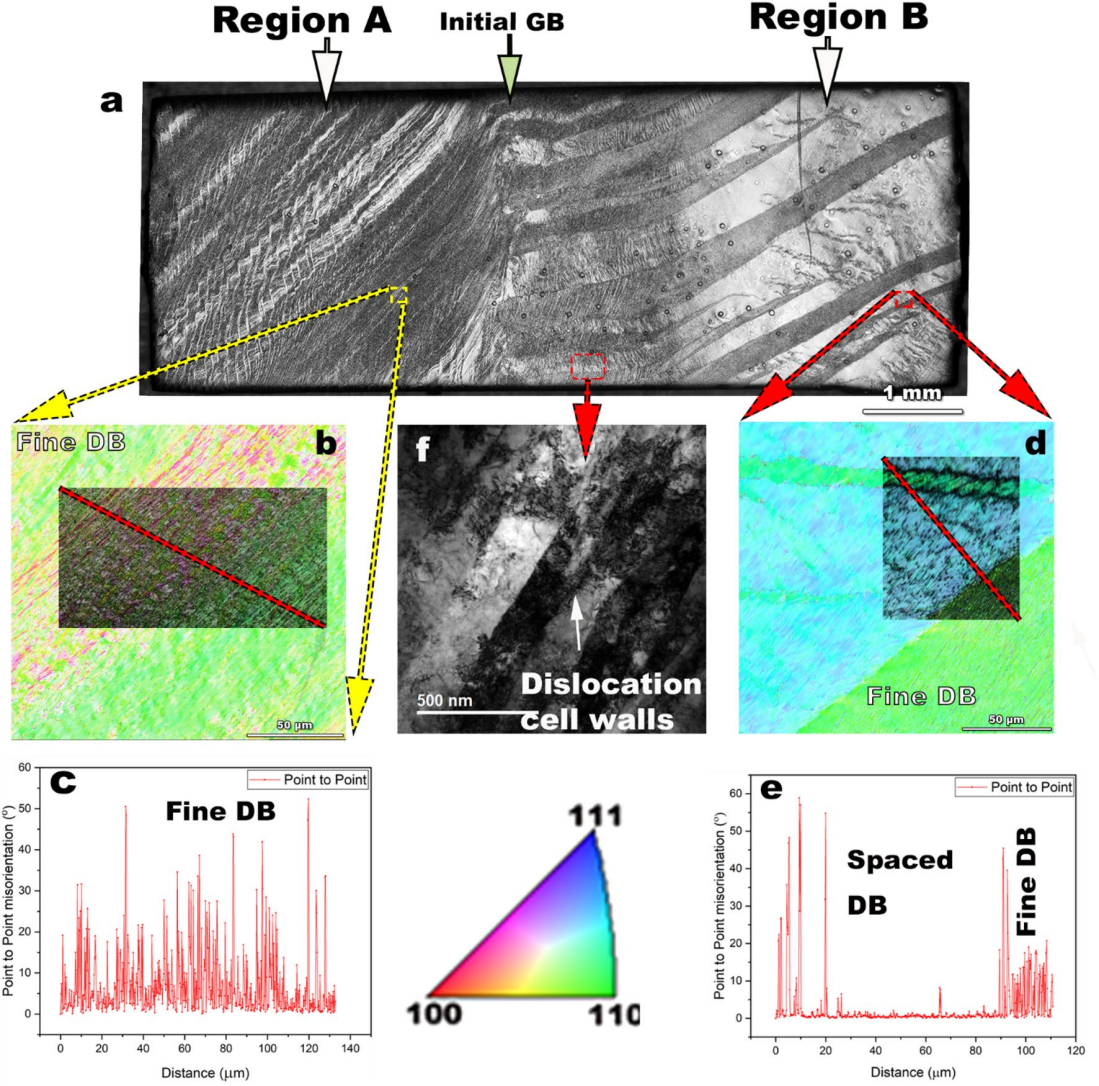
The simple shear deformation of the Nb bi-crystal produces differences in deformation structures depending on initial crystal orientations as shown by (**a**) a light microscope image of the polished and lightly etched surface indicating deformation band (DB) structures that occur in high purity Nb, (**b**, **d**) which are inverse pole figure (IPF) maps of regions showing predominant differences in DB structure between Region A, and Region B, and c) and (**e**) which show the variations in misorientation angles as a function of location, marked by the red lines in IPF. Uniform variations of misorienation in Region A are distinguished by alternating sparse-fine DB in Region B. The legend describes the color codes for grain orientation. The IPF’s were generated using the TSL OIM Analysis (Version 7.0), link: https://www.edax.com/products/ebsd/oim-analysis.

Deformation by simple shear of an initial Nb bi-crystal with initial Grain A, Euler angles—338.2°, 40.3°, 69°, and Grain B, Euler angles—313.2°, 71.3°, 25.7° leads to spatial variations in the structure depending on the initial orientation^28^. Figure 1 depicts the sample after deformation, and chemical etching of the surface after deformation indicates the initial grain boundary (GB) separating the grains. Buffered chemical polishing (BCP) is frequently used to produce polished cavity surfaces but etches grains unevenly, for our samples, we apply a light buffered chemical polishing (BCP) to reveal surface grain domains while obtaining a high-quality initial surface. The deformed grains designated as region A corresponding to initial grain A, region B corresponding to initial grain B. The distinct microstructural regions are clearly visible in the light microscope image shown in Fig. 1(a). Region A consists of a finer deformation band (DB) structure over a wide area, as indicated in Fig. 1(b), and the corresponding point to point misorientation angle values between adjacent regions is shown in Fig. 1(c). In contrast, Fig. 1(d) shows region B consisting of a coarse DB structure matrix with a very low point to point misorientation between strips of finer DBs. The complex deformation structures on a microscopic scale consist of characteristic dislocation network structure with well-formed cell wall structures, as indicated in Fig. 1(e). This cell structure is clearly visible in in-plane bright field (BF) image of transmission electron microscopy (TEM), Fig. 1(f).

**Figure 2:**
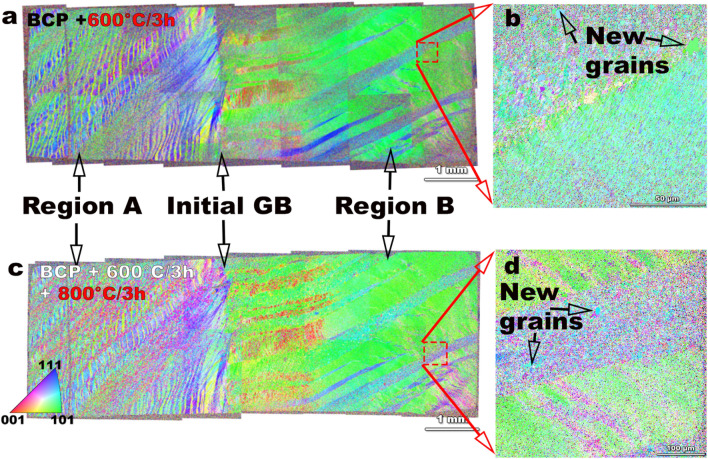
SEM-EBSD IPF maps of the entire bi-crystal after deformation and heat treatment at (**a**) 600 °C/3 h, and (**c**) after 800 °C/3 h. Extended high resolution IPF maps show some new grains that emerge from the deformed matrix after 600 °C/3 h (**b**), and 800 °C/3 h (**d**). There are no major texture variations or microstructure changes up to 800 °C/3 h heat treatment. Significant grain growth processes are not active even at 800 °C/3 h heat treatment condition in this study. The legend decribes the color codes for grain orientation. The IPF’s were generated using the TSL OIM Analysis (Version 7.0), link: https://www.edax.com/products/ebsd/oim-analysis.

Subsequent heat treatment of the deformed bi-crystal at 600 °C/3 h, 800 °C/3 h, and 1000 °C/3 h led to changes in the microstructures. Figure 2 compares the plane view EBSD-OIM parallel to the surface normal after the 600 °C/3 h and 800 °C/3 h heat treatments, showing no major changes in macroscopic grain orientations or grain morphological structures in either region A or B. The OIM IPF images shown in Fig. 2(a) after 600 °C/3 h indicate that region A with a finer band structure shows variations with alternating bands of {101} and {111} orientations. Region B, on the other hand, consists of large laths of {101} bands with {111} orientation bands in between. Interestingly, some new crystal orientations began to appear from the deformed microstructure. There are slight variations from Fig. 2(a) in micro-texture components and microstructure after 800 °C/3 h heat treatment (Fig. 2(c)). In region A, the initial {101} bands likely re-oriented towards {100} orientations and region B indicates grain growth from already recrystallized grains with a preference for {100} orientations. Region B consists of a larger fraction of {101} orientations with some signs of recrystallization. These results do not imply significant variations in microstructure, perhaps indicating slight recovery rather than recrystallization by grain growth.

**Figure 3:**
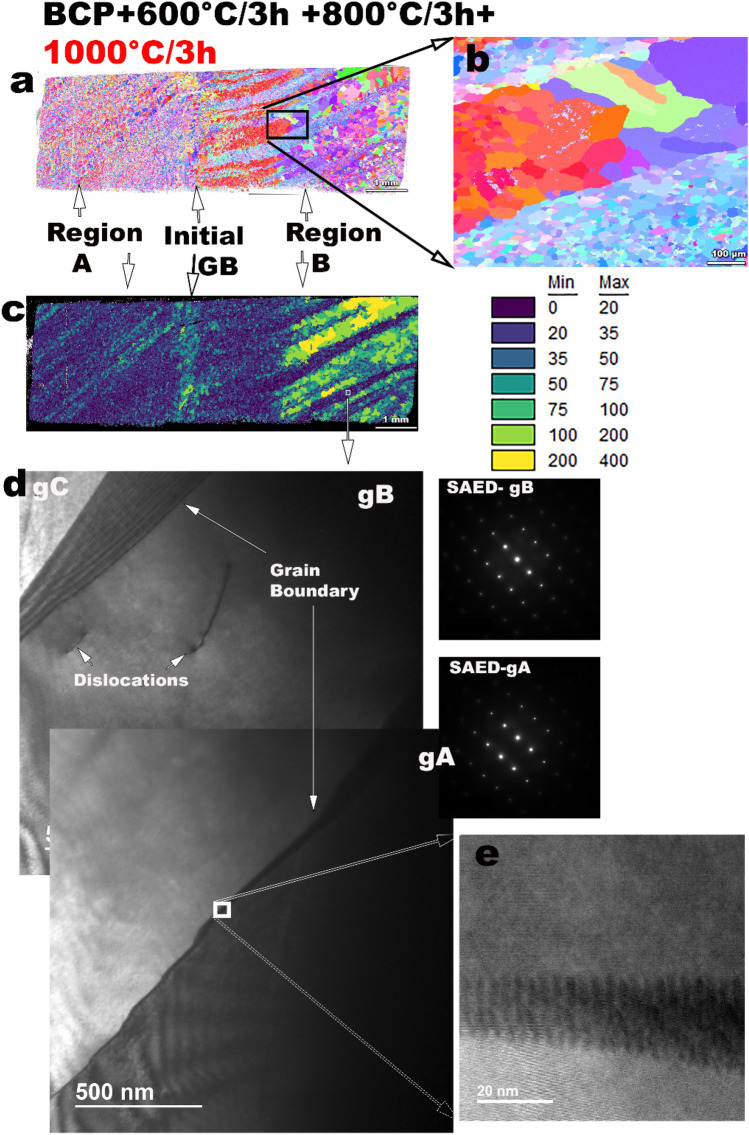
Microstructure development after 1000 °C/3 h heat treatments as indicated by IPF maps as well as TEM microscopy: (**a**) IPF map of the entire sample indicates growth textures differ from deformation textures, and formation of mostly a 30–50 µm fine grain (FG) microstructure in Region A, and (**b**) duplex large grain (LG)-fine grain (FG) microstructure characteristic of Region B, (**c**) grain size distribution map from (**a**) with the color code for grain size in μm, (**d**) bright field (BF) STEM with corresponding selected area electraon diffracton (SAED) patterns of the fine grains from Region B at z = [110] zone axis, indicating very few dislocations and no dislocation networks, as well as clean grains as observed in a representative high magnification image (**e**). The IPF’s were generated using the TSL OIM Analysis (Version 7.0), link: https://www.edax.com/products/ebsd/oim-analysis.

There is a dramatic difference in the texture and microstructure after the 1000 °C/3 h heat treatment, as shown in Fig. 3(a). Region A consists of a uniform fine grain microstructure with grain sizes of the order of 35–50 µm as indicated by the color-coded grain size map of the microstructure in Fig. 3(c). Microstructures in region B develop into a duplex grain structure as shown in Fig. 3(b), with fine grain bands of grain size < 50 µm surrounded by larger grain size bands of the order of 100–400 µm. The orientations after grain growth are completely different from those after the 600 °C and 800 °C heat treatments. The pre-dominant orientations after the recrystallization by grain growth processes are now {100} and {111} in different regions. The grain orientation color code is the same as Fig. 1 and Fig. 2. Figure 3(d) shows the bright field (BF) images of scanning transmission electron microscopy (STEM) performed on the fine grain band in region B. Some of the individual dislocation lines were clearly visible in region B without dislocation networks in the areas surveyed. The diffraction patterns indicate BCC z = [110] zone-axis. The observed microstructures were free of precipitates and contaminants, and the diffraction patterns show no residual evidence of Nb hydride or oxide precipitations in this room temperature investigation. The extended grain boundary contrast due to zone-axis TEM imaging, at the top left of Fig. 3d) indicates that the grain boundaries in this duplex grain structure are produced by the stacking of dislocations. From the TEM images, we conclude that after 1000 °C, there is recrystallization and growth of new grain orientations in Nb, replacing the deformation microstructure even in the fine grain regions.

In summary, the initial grain orientation plays a significant role in the deformation structures, and it varies spatially. During successive heat treatments, recovery processes are supposed to be pre-dominant at a temperature range of 600–800 °C. After 1000 °C/3 h heat treatment, recrystallization and significant grain growth have occurred in the deformed Nb. A characteristic change in the textures before and after 1000 °C/3 h is evident. However, the microstructure is non-uniform spatially. The deformation band region consists of fine grains (30–50 µm), whereas the region with widely spaced deformation bands develops a duplex microstructure with large grains of the order of 100–400 µm, and adjacent 30–50 µm fine grain size bands.

### Detection of magnetic flux penetration and trapping by Magnetic Optical Imaging (MOI)

**Figure 4:**
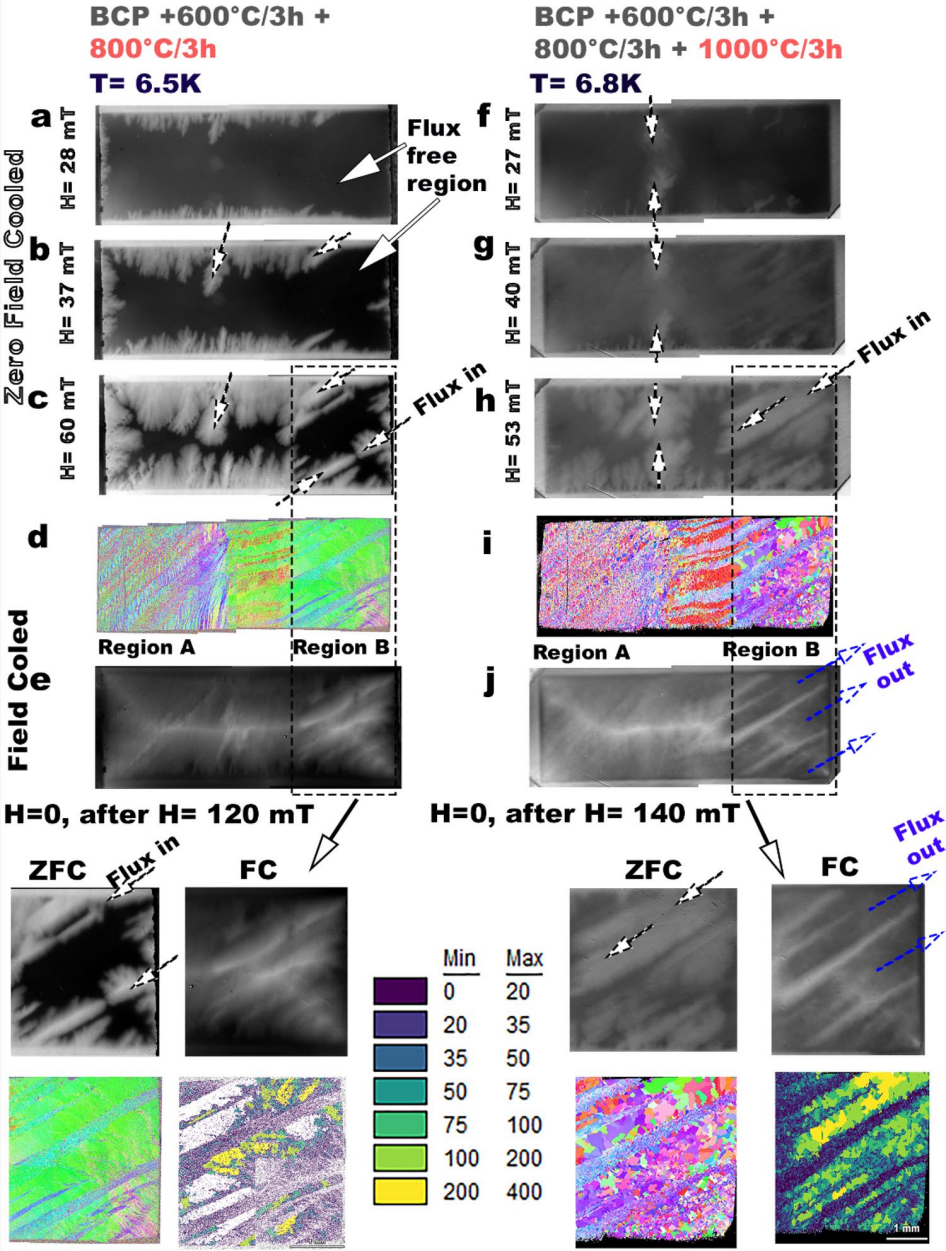
MOI images showing the flux flow characteristics in Nb after different heat treatments that show significant microstructure variations, (**a**–**c**), and (**f**–**h**) indicate flux flows into Nb at T (6.5–6.8 K) < T_c_ (9.2 K) of Nb with increasing magnetic fields starting from an initial fully superconducting state, based on zero-field cooled (ZFC) mode. (**d**, **i**) represent the full cross-section microstructure as a reference from EBSD-OIM, and (**e**, **j**) trapped flux images based on field cooled (FC) mode, indicating flux exit or expulsion from select regions of large grain (LG: 100–400 µm) microstructure, and flux trapping along fine grain (FG: < 100 µm) regions. The dark regions in the MOI images represent flux free regions. In an ideal superconductor, external magnetic fluxes are completely expelled after removing the external field. The IPF’s were generated using the TSL OIM Analysis (Version 7.0), link: https://www.edax.com/products/ebsd/oim-analysis.

To understand the microstructural influence on penetration and trapping of magnetic flux, we performed MOI along with deformation and heat treatments. The results presented here are after 800 °C/3 h and 1000 °C/3 h at temperatures 6.5 and 6.8 K, respectively, where Nb (T_c_ ~ 9.2 K) is in the fully superconducting state (supplementary section Figure S1contains results of sample after deformation + etching, and 600 °C/3 h heat treatment). Figure 4 shows a series of MO images of the bi-crystal after 800 °C/3 h and 1000 °C/3 h heat treatment after undergoing zero-field cooling (ZFC) where the sample is initially cooled and is in a fully superconducting state, then, external magnetic fields are slowly applied, and magnetic flux lines start penetrating along the specific areas, as indicated by the arrows in Figs. 4(a–c) and (f–h). The center black strip in MO images in Fig. 4(a–c and f–h) is a flux free region without Abrikosov vortices. The width of the flux free region correlates with the critical current. Orientation maps of the sample are provided as a reference in Figs. 4(d,i). The flux initially penetrates around the original GB region as the magnetic field is increased. A dendritic-like flux penetration is observed after the 800 °C/3 h HT, and the flux penetration further diffused into the sample after the 1000 °C/3 h HT. The spatial flux penetration characteristics of region A and region B are different and further changes are observed with heat treatment temperature. From Fig. 4(a–c) and (f–h) we see that the large grains admitted flux earlier than the fine-grain regions did. A microstructure-related pattern of flux penetration is very apparent in region B. In Fig. 4(c), there is an absence of flux penetration in region B after the 800 °C/3 h where the fine grain band is located, whereas, in Fig. 4(h) the same region shows flux penetration when the grains have grown to about 100–400 µm after 1000 °C/3 h. In region A there are fewer variations, which is consistent with the lack of variations in microstructure after heat treatments.

The bulk magnetic flux trapping behaviour of the bi-crystal was determined in the field cooled (FC) mode, where the external magnetic field was applied at T > T_c_, and cooled below T_c_. Removing the external field at T < T_c_ returns the sample to the superconducting state. In an ideal superconductor a complete flux expulsion occurs, and the whole sample will be in superconducting state T < T_c_, and H = 0. The remnant field images in Figs. 4(e,j) show the regions where flux trapping is more favorable. The dark regions indicate superconducting region where flux has been expelled and the light regions show where magnetic field has been trapped. Comparing the MOI image in Fig. 4(e) with the microstructure in Fig. 4(d) we see that the fine grain (FG) band structures of region B are susceptible to flux-trapping, while some of them did not preferentially admit flux penetrations at ZFC mode (Fig. 4c). Figure 4(j) shows that Region B with 100–400 µm of large grains that exhibited preferential flux penetration in the ZFC mode, completely expelled magnetic flux. Flux is trapped in the finer grain regions where the grain size is of the order of 30–50 µm. The insert in Fig. 4, illustrates the correlation of flux entry, and flux expulsion with grain size. The classic rooftop pattern was produced for both the FC MO images due to field enhancement from the rectangular sample shape. MOI of region A shows subtle variations in trapping with microstructure, visible as streaks, which coincide with small variations in the grain size.

In summary, flux penetration occurs from the edges of the deformed and heat treated Nb sample and follows the critical state model^56^. Flux penetrates into the larger grain (100–400 µm) earlier than fine grain regions (30-50 µm). Most importantly, flux is completely expelled from the large grain regions (100–400 µm), whereas most of the flux-trapping occurs in the fine-grain regions (< 50 µm).

### Pinning force variations with heat treatment

**Figure 5:**
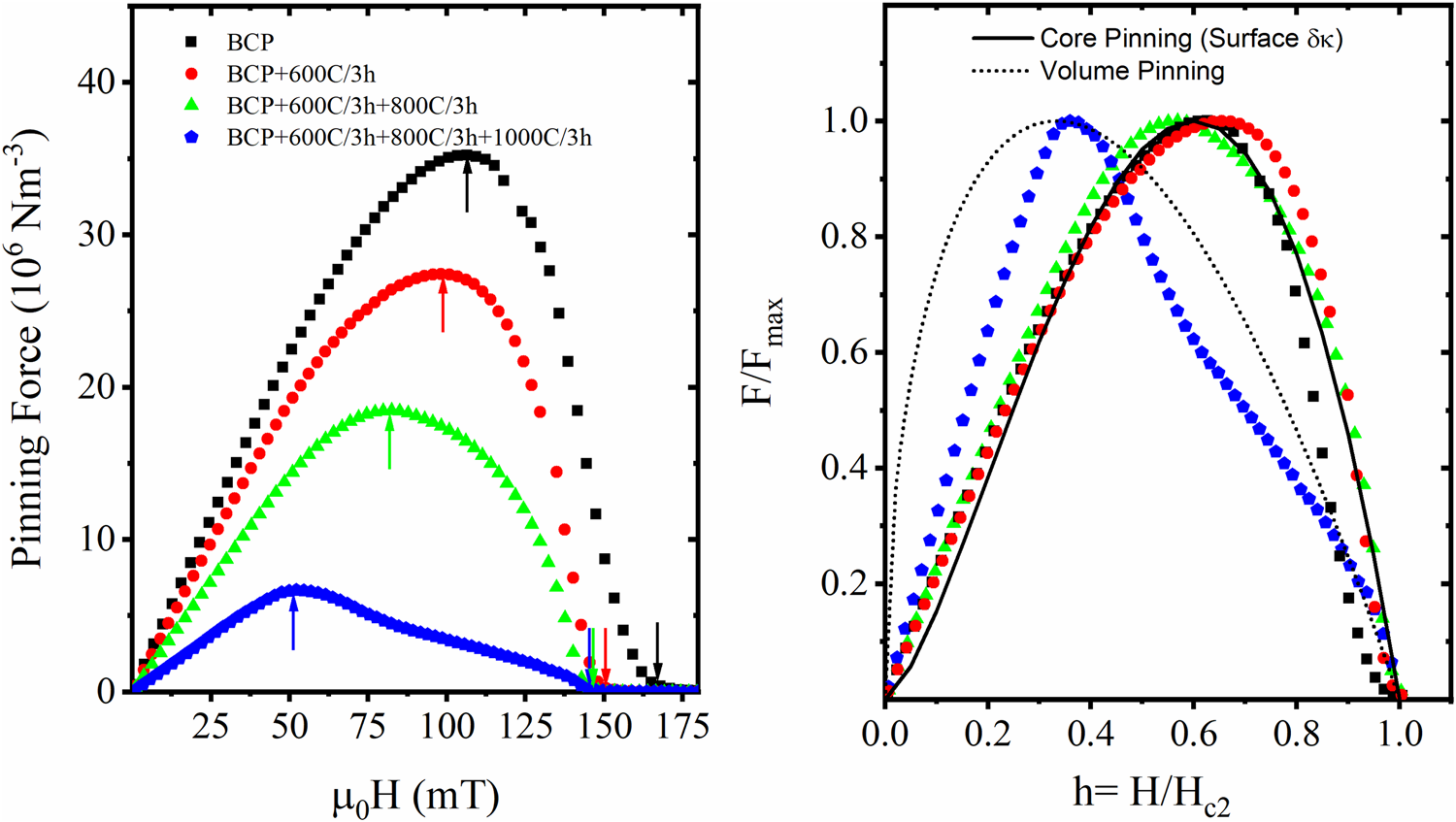
Pinning force curves at T = 7.0 K, comparing the bulk pinning force behavior in the as-received state and after heat treatment: (**a**) Pinning force as a function of reduced field H = μ_0_H (mT) indicates that pinning force magnitude drops significantly with heat treatments, (**b**) normalized pinning force plot of *F*_*p*_*/ F*_*p,max*_ versus h *(H/H*_*c2*_) are similar in the as deformed, after 600 °C/3 h, and 800 °C/3 h heat treatments, and follows the core pinning related to local variation in *κ* as described by Dew-Hughes, whereas after 1000 °C/3 h the variations do not fit a clear model and collective pinning mechanisms are being further investigated.

DC magnetic hysteresis loops were measured at ~ 7 K for Nb bi-crystal samples from the deformed state with variations of heat treatment (600–1000 °C/3 h) in order to determine the bulk pinning to compare with the results of the MOI study. The pinning behavior of the Nb is plotted as a function of external magnetic fields at 7 K as shown in Fig. 5(a,b). The pinning force (*F*_*p*_) in Nb is weak as indicated by the low bulk *F*_*p*_ magnitude of the order of several MN/m^3^ in all samples. However, the maximum pinning force (*F*_*p*_) is 34 MN/m^3^ for the deformed sample and decreases progressively and ultimately by order of magnitude to 6 MN/m^3^ after the 1000 °C/3 h heat treatment. This expected reduction is due to the removal of pinning sites by thermal treatment. The irreversibility field (*H*_*irr*_) also shows a similar decreasing trend from the as-deformed and through the series of heat-treated samples, which is consistent with the decrease in flux pinning as heat treatment temperature is increased. A plot of normalized pinning force against a reduced field is shown in Fig. 5(b). There are no significant changes in the pinning force behavior between the as-deformed followed by BCP’ed sample versus the samples followed by 600 °C/3 h, and 800 °C/3 h heat treatments, however, applying the final 1000 °C/3 h heat treatment produces a significant change. The pinning mechanism corresponds to core surface pinning, as indicated by the Dew-Hughes pinning model^29^. After the 1000 °C/3 h heat treatment, the pinning force maximum shifts to a lower reduced field with the peak of the pinning force corresponding to volume pinning^29^ but the overall pinning mechanism could arise from collective contributions of surface, magnetic and volume pinning.

## Discussion

Shear deformation is a characteristic of most common material processing operations and is very relevant for SRF Nb where the Nb sheets are fabricated by rolling and successive heat treatments to form polycrystalline material^30^. The simplest polycrystalline material that can be investigated is a bi-crystal. Even though a uniform deformation may be applied to an Nb sheet across its thickness, the microstructure after deformation and heat treatment is highly heterogeneous and is sensitive to the material history. The microstructure is influenced by the initial orientation, deformation history, heat treatment, and annealing cycles that have taken place to reduce the original Nb ingot to the ~ 3 mm sheet thickness typically used for cavity fabrication. Thus, understanding these individual steps and their interplay is crucial for the future development of low loss resonators that involve time-varying fields.

Specifically, the deformed microstructure forms low energy dislocation structures^31^ with the development of cell wall structures after deformation, as seen in Fig. 1(f), characteristic of medium to high energy stacking fault energy (SFE) materials^32^. Niobium with an SFE ~ 40^33^–150^34^ mJ/m^2^ is considered to be medium–high SFE material and forms cell wall structures after 25% rolling deformation^35^. In this study, the Nb deformation corresponds to about 67% rolling reduction (RR)^28,36^, and the texture evolution in the Nb bi-crystal also follows the {110}||surface normal, shear deformation texture as observed in simple shear processed bcc metals^37^, as observed after the low-temperature heat treatments 600 °C, 800 °C/3 h. Variations in microstructure evolution based on initial crystal orientation affecting deformation characteristics in Nb have been previously observed, and the formation of variations in DB’s are related to multiple-slip possibilities for certain orientations to maintain strain compatibility^38,39^. Differences in recovery and recrystallization behavior are also observed in cold-rolling deformation of Nb. In a study where an initial Nb orientation {110}<100> is cold rolled to 80% RR, different regions of the grains split into widely spaced DB^40^, a feature that we observe in region B after deformation in Fig. 1. Heat treatments provide energy to diffuse on an atomic scale that causes annihilation of point and dislocation-based defects. However, for full recrystallization a critical amount of mechanical strain is normally required, as well as a sufficiently high temperature to surpass the activation energy barrier for large scale atom diffusion. A rule of thumb for recrystallization is a minimum cold work with ~ 70% RR, and at a critical temperature of the melting point of the metal (T_m_) in Kelvin scale divided by 3^41^. The melting point of Nb is 2750 K, and T_m_/3 is 916 K (~ 650 °C). According to this rule of thumb calculation, there should be some recrystallization around 600–800 °C. However, recrystallization by grain growth is relatively limited, as observed in Fig. 2. There is evidence that low deformation strains^42^ and up to 70% RR^43^ is not sufficient to complete the recrystallization processes in high purity Nb (RRR: residual resistivity ratio > 250). Our study shows that high-temperature heat treatment of 1000 °C/3 h is needed to obtain recrystallization and growth. The reason for the formation of the duplex microstructure needs further investigation since we observed no obstacles to the movement of GBs due to precipitates or hydrides phases or residual dislocations in the TEM images in Fig. 3. The duplex microstructure indicates that there are regions that undergo abnormal grain growth, creating 100–400 µm size grains, whereas there are other grains of the order of 30–40 µm. Duplex microstructures could occur due to impurity, or solute segregation at GBs preventing GB mobility of some grains leading to polygonization like fine grains, whereas other grains grow uninhibited. The other possibility is that in the presence of variations in microstructure between regions, there are deformed regions which have a higher number of potential nucleation sites, leading to finer grain sizes than other regions. Heterogeneous nucleation is likely a candidate mechanism for duplex microstructure in this study since there are fine grains in region A after 1000 °C as shown in Fig. 3. The likelihood of fine micro-band structure, as shown in Fig. 1(b,c) increases the opportunities for heterogeneous nucleation with multiple potential nucleation sites availability, and hence GB mobility is impeded by the competitive growth of several grains. The variations in the grain size corresponding to the DB structure after deformation (Fig. 1) point towards history and deformation mode being critical components that need to be controlled to obtain optimum and reproducible microstructures. No segregation of interstitials was found in fine grain regions as shown in TEM images Fig. 3(d,e), however, solute segregation of interstitials like oxygen, carbon, and nitrogen cannot be completely ruled out and would require verification by atom probe tomography or by other techniques.

The strong relationship between the microstructure and the flux penetration and flux trapping is evident in Fig. 4. Most strikingly, on increasing the magnetic field beyond *H*_*c1*_ (6.5 K) of 10 mT, variations in the flux penetration are clearly related to the microstructure of the sample as determined by EBSD-OIM. In the sample after 800 °C/3 h the flux penetration is in the dendrite-like form from the outer edges. In previous studies^44–47^, dendritic flux penetration showed current instability pattern in the form of avalanches due to local overheat in the edges along the perimeter of thin-film tapes, however, this is not the case for our bulk sample. In our samples the flux penetration follows the critical state model. The pinning centers after the 800 °C heat treatment are predominantly related to the deformation structures that are present after recovery and some recrystallization, which could also include dislocation structures. The flux entry and flux exit behavior after the 800 °C HT could be dominated by dislocation structures, and flux expulsion is likely pre-dominant throughout the sample. This result clearly suggests that flux expulsion would be poor when there are dislocation structures in SRF Nb. This is consistent with poor flux expulsion that was observed when an Nb cavity was deformed by mechanical tuning^23^.

Changes in the flux trapping and penetration behavior that we observe after the 1000 °C/3 h heat treatment indicate the influence of recrystallization by grain growth on flux flow into Nb. The diffuse pinning confirms the reduction in pinning centers with heat treatment. The most notable finding is the significant variation in the flux flow with recrystallization at 1000 °C/3 h heat treatment, where the only microstructural features are grain boundaries. From the sequential ZFC (surface sensitive) MOI images of the flux flow in Fig. 4(f–h), it is clear that flux begins to enter easily through the large grain regions possibly due to a significant reduction in GB obstacles compared to the high GB densities in fine-grain regions.. However, in field-cooled (FC) MO images (sensitive to bulk structural properties), once the field is removed, the fine grain regions are more susceptible to flux trapping and expel flux poorly, which is consistent with a larger number of pinning centers obstructing the flux flow path in the fine grain regions. Previous MOI studies have shown the impact of hydride formation on flux penetration and trapping, as well as the importance of microstructural features such as low angle grain boundaries^48^. In the heat treated samples, there is no evidence of a high density of non-GB pinning centers dislocations and precipitates. However, there is a correlation between grain boundary density and flux trapping and poor expulsion in these samples. These results show that a greater driving force is needed to expel flux from 30 to 50 µm fine-grain regions compared to 100–400 µm large grain regions. The relative orientation of the magnetic field and the GBs has been shown to be important for the pinning properties of SRF Nb for dc field flux trapping by previous experiments on current flow transport measurements^49–51^ and MOI imaging. Higher GB density could provide more avenues for the magnetic field-GB orientation disruptions, which are also hypothesized to affect the electronic states of Nb^52^.

Bulk magnetization measurements can capture the variations in pinning force and mechanism, as shown in Fig. 5. The systematic drop in maximum pinning force magnitude from 34 to 17 MN/m^3^ from as deformed condition to 800 °C/3 h heat treatment suggests that defect density reduction can be tracked with the maximum magnitude of the pinning force curves and the value of *H*_*irr*_ as shown in Fig. 5(a). The normalized pinning force curves in Fig. 5(b) indicate that there are no changes in the pinning force mechanism between a deformed sample and 800 °C/3 h and can be fit with the vortex core surface pinning model^29^. After the 1000 °C/3 h heat treatment, the significant shift of the pinning force curve to the lower field suggests a change in the pinning mechanism. The change in the shape of the curve indicates that a combination of pinning mechanism is possible due to the duplex microstructure. The decreased pinning force also relates to the contrast differences in the MO images between Fig. 4c,h due to the reduction of critical current with increased heat treatment temperature. The use of pinning force curves could be a promising technique to provide additional insights into the flux trapping behavior using coupons samples as a testbed, especially when combined with theoretical modeling.

The outcome of this work suggests that particular attention should be given to the recovery and recrystallization of polycrystalline Nb as both dislocation structures and recrystallized grains < 100 µm can contribute to flux trapping. This study shows that duplex microstructures with fine grain bands alternating with large-grain regions can appear even after a 1000 °C/3 h annealing and contribute significantly to flux trapping in high purity Nb. To achieve reproducible SRF properties, developing uniform Nb microstructures of optimum grain size that minimize flux trapping is essential. Further work on Nb microstructure development, and superconducting property correlations involving the upstream Nb sheet manufacturing process are currently being pursued.

## Methods

### Description of raw material

**Figure 6:**
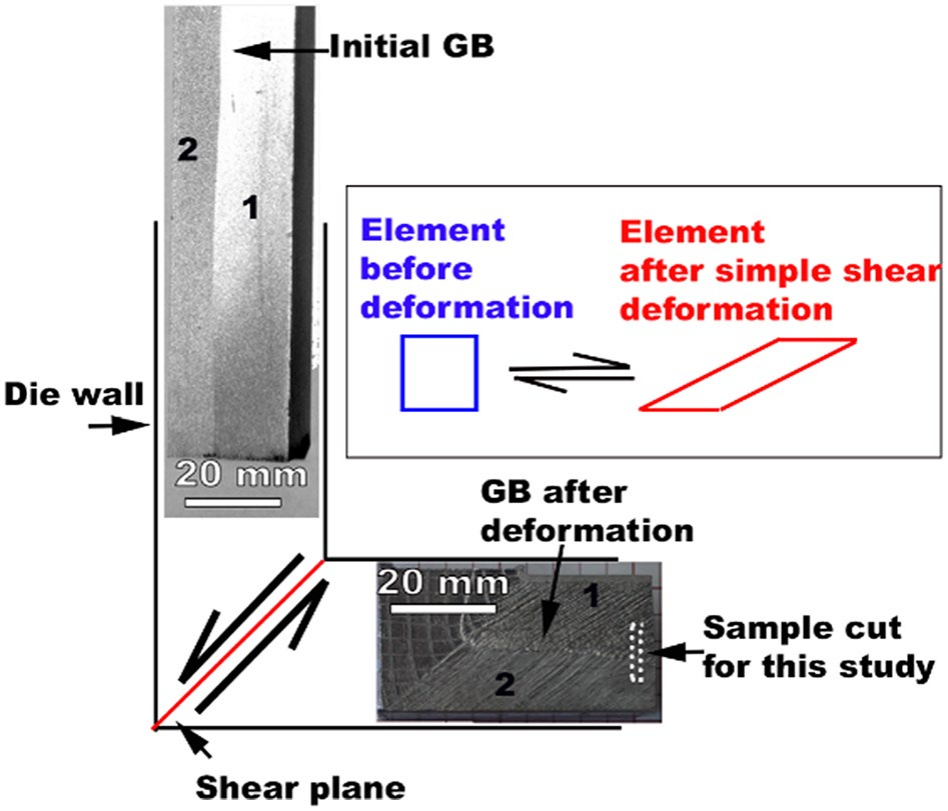
Schematic of simple shear deformation of a Nb bi-crystal. The deformation leads to a shear strain of 1.14, which corresponds to an equivalent plastic strain of a rolling reduction deformation.

The raw-material used in this study was an electron beam (EB) melted large grain Nb ingot obtained from Niowave. The Nb billet had a starting residual resistivity ratio (RRR) of 176 ± 10. The chemical analysis of the material as performed by the vendor indicated a Ta content of ~ 550 ppm, oxygen ~ 97 ppm, nitrogen ~ 34 ppm, carbon < 30 ppm, and hydrogen < 10 ppm. A bi-crystal of dimensions 50 × 50 × 250 mm^3^ was cut from the ingot. The bi-crystal underwent a single pass deformation by equal channel angular extrusion (ECAE). A single ECAE pass leads to a shear strain of 1.14. The schematic in Fig. 6 shows the uniform simple shear deformation experienced by the bi-crystal used in this study, more details about processing and deformation characteristics are provided elsewhere^28^. A sample of nominal dimensions of 10 × 3 mm^2^ was cut across the two grains including the deformed initial GB as shown in Fig. 6. The sample was initially polished flat and lightly etched with a traditional BCP etch- HF: HNO_3_: H_3_PO_4_ in the ratio 1:1:2. To avoid any subsequent introduction of hydrogen, the sample did not undergo any further mechanical polishing after the initial polishing step. The sample investigated in this study started out at an initial sample thickness of ~ 1 mm, and was polished down to 0.5 mm with the last step that involved 0.05 μm colloidal silica polishing to obtain a mirror-like surface finish.

### Sequential processing and sample characterization

MOI uses the concept of a double Faraday effect to optically image the behaviour of a superconductor in terms of flux penetration and flux trapping^53^. It is a specialized technique used to identify defects in superconductors and variations in superconducting properties, the method has been well developed for Nb^48,54,55^. MOI was performed in the zero field cooled (ZFC) mode where the Nb sample was cooled below *T*_*c*_ (~ 9.2 K) in the absence of external magnetic field. At a stable temperature below *T*_*c*_, the field was slowly increased beyond the lower critical field (*H*_*c1*_). When *H*_*c1*_ < *H* < *H*_*c2*_ the flux penetration into the sample is observed using a polarized light microscope with a garnet indicator film. Once the sample reaches a field beyond the upper critical field (*H*_*c2*_) the flux completely penetrates the sample.

In order to investigate bulk flux trapping characteristics, the sample is investigated in an imaging condition called the field Cooled (FC) mode. In this mode, a magnetic field is applied at a temperature greater than *T*_*c*_, where the magnetic field is sufficient to fully penetrate the sample. The sample is then cooled to below *T*_*c*_, and the external field is turned off. In an ideal superconductor a complete flux free state with full flux expulsion is obtained. However, if there are regions with variations in superconductivity or defects which traps the magnetic field, can be imaged by the MOI technique. MOI in the ZFC and FC modes was performed on the sample after each heat treatment. All sample heat treatments were carried out in a dedicated furnace for SRF cavity heat treatment at Jefferson Lab facility.

Scanning electron microscopy was performed on the sample after each heat treatment using a field emission Zeiss-ESB scanning electron microscope. Orientation image mapping (OIM) was performed in this microscope using a high speed Hikari camera and EDAX EBSD software for OIM analysis (Version 7). High resolution microstructural evaluations were performed using a JEOL ARM 200 F transmission electron microscope (TEM) operated at 200 kV. The TEM specimens were prepared by using focused ion beam (FIB) milling. Prior to FIB milling a protective layer of platinum (3 μm wide, 2 μm long, and 1 μm thick) was deposited on the sample to preserve the surface of area of interest during high energy gallium ion (Ga +) bombardment. A lamella (2 × 20 × 12 μm^3^) was lifted and placed on a TEM grid. The lamella was subsequently milled on both sides to form a thin window sufficiently electron transparent (100 nm) to allow detailed images to be recorded. It was observed that the Ga ion bombardment can cause damage on the surface layers which could be easily removed by chemical removal using the BCP 1:1:2 mixture.

### Magnetization measurements

For dc magnetization measurement, a sample with nominal dimension of 4.94 × 1.91 × 0.74 mm^3^ was cut from the bi-crystal using a diamond saw. The isothermal dc magnetization measurement was performed using a 5 T Quantum Design magnetic property measurement system by applying external dc magnetic field perpendicular to the 4.94 × 0.74 mm surface. The pinning force was determined by the relation, *F*_*p*_ = *B* × *J*_*c*,_ where, *B* is the external applied field and *J*_*c*_ the critical current density which for the current condition is given by *J*_*c*_ = *ΔM/N*_*d*_, where *ΔM* is the difference in magnetization for external field *B* during increasing and decreasing field on hysteresis loop, and *N*_*d*_ is a geometric factor related to the sample dimension^56^. The magnetization measurements conducted here are made with the magnetic field perpendicular field to the imaged surface, which is the same field orientation as the MOI measurements. The flux trapping issues of primary concern in SRF Nb cavities are related to ambient magnetic fields, which can be randomly oriented.

## Supplementary Information

Supplementary Information 1.

## Data Availability

All the data presented here in this paper will be available based upon request.
