# Supplementary material for: Direct evidence of microstructure dependence of magnetic flux trapping in niobium
Source: Sci Rep. 2021 Mar 8;11:5364. doi: 10.1038/s41598-021-84498-x (PMC7940417; doi:10.1038/s41598-021-84498-x)
Supplement: Supplementary file 1 — Supplementary Information 1. [file 41598_2021_84498_MOESM1_ESM.docx]

Direct evidence of microstructure dependence of magnetic flux trapping in niobium

Shreyas Balachandran^1,*^, Anatolii Polyanskii^1^, Santosh Chetri^1^, Pashupati Dhakal^2^, Yi-Feng Su^1,3^, Zu-Hawn Sung^1,4,^ and Peter J. Lee^1^

^1^Applied Superconductivity Center, NHMFL-FSU, Tallahassee, Florida 32309, USA
^2^Thomas Jefferson National Accelerator Facility, Newport News, Virginia 23606, USA

^3^Oak Ridge National Laboratory, Oak Ridge, Tennessee 37830, USA

^4^Fermi Lab, Batavia, Illinois 60510, USA

^*^shreyasb@asc.magnet.fsu.edu

**Supplementary Section**

MOI imaging was performed on the as deformed sample after buffer chemical polishing (BCP) to systematically track the flux penetration behavior and the flux expulsion behavior. In the deformed state and after 600°C/3h there are no major changes in the microstructure hence the qualitative variations in the flux flow behavior do not vary as seen in Figure A1.


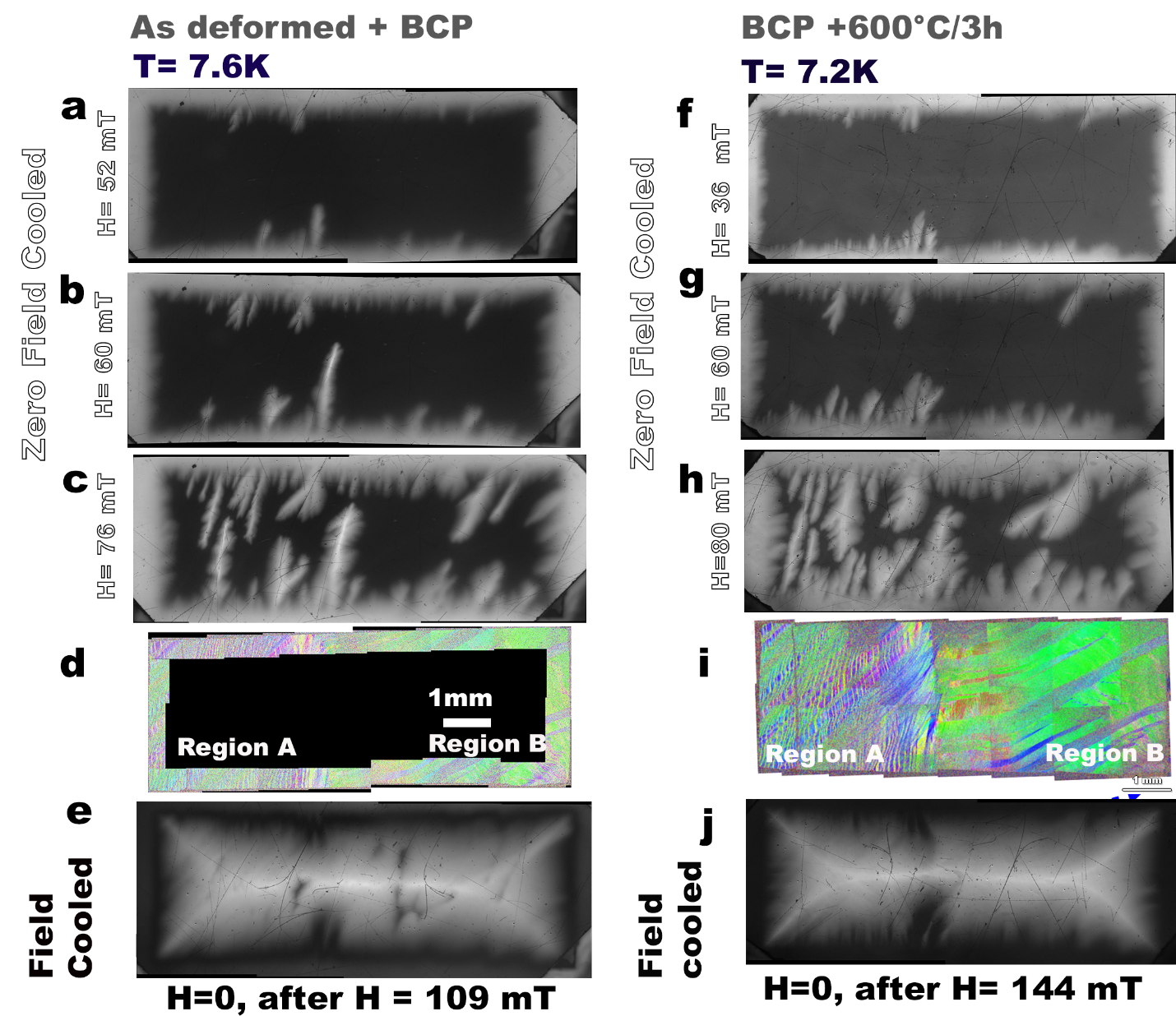


MOI images showing the flux flow characteristics in Nb after deformation andafter 600°C/3h heat treatment., a)-c), and f)-h) indicate flux flows into Nb at T (7.2-7.6 K) < T_c_ (9.2 K) of Nb with increasing magnetic fields starting from an initial fully superconducting state, based on zero-field cooled (ZFC) mode. d), represents the microstructure of the outer edge, whereas (i) represents a full crossectional microstructure, and e), j) trapped flux images based on field cooled (FC) mode, indicating some flux exits or is expulled from the grain boundary region, but most of the flux is trapped.
